# Heat Shock Proteins at the Crossroads between Cancer and Alzheimer's Disease

**DOI:** 10.1155/2014/239164

**Published:** 2014-07-24

**Authors:** Hao Wang, Meng-Shan Tan, Rui-Chun Lu, Jin-Tai Yu, Lan Tan

**Affiliations:** ^1^Department of Oncology, The Affiliated Hospital of Qingdao University, Qingdao 266003, China; ^2^Department of Neurology, Qingdao Municipal Hospital, College of Medicine and Pharmaceutics, Ocean University of China, Qingdao 266003, China; ^3^Department of Neurology, Qingdao Municipal Hospital, School of Medicine, Qingdao University, Qingdao 266071, China

## Abstract

Heat shock proteins 70 and heat shock proteins 90 (Hsp70/90) have been implicated in many crucial steps of carcinogenesis: stabilizing oncogenic proteins, inhibiting programmed cell death and replicative senescence, induction of tumor angiogenesis, and activation of the invasion and metastasis. Plenty of cancer related proteins have the ability of regulating the expression of Hsp70/90 through heat shock factor 1. Cancer and Alzheimer's disease (AD) have plenty of overlapping regions in molecular genetics and cell biology associated with Hsp70/90. The Hsp70, as a protein stabilizer, has a cellular protection against neurodegeneration of the central nervous system, while Hsp90 promote neurodegenerative disorders indirectly through regulating the expression of Hsp70 and other chaperones. All these make existing anticancer drugs target Hsp70/90 which might be used in AD therapy.

## 1. Introduction

The occurrence of cancer and Alzheimer's disease (AD) increases exponentially with age, and growing evidence suggests an inverse relationship between the risks of the two disorders according to the epidemiological data from the literatures [1–4]. Cancer characterized by unregulated cell growth and AD denoted by neuronal cell death could be hardly found any common in the first glance, but the more molecular genetics and cell biology of cancer and AD we discovered, the greater the overlap between the two diseases appears, especially in the drug target region. Based on previous studies, proteins regulating cell death and survival play a key role in the development of the two diseases. Cancer is caused by multigene mutations, leading to increased oncoprotein expression and sustained activation of cell proliferation signals. The tumor cells with increased protein load accompany transformation and the inherent instability of many mutant proteins. And AD, a “protein folding” disease, has extraneuronal deposits of amyloid-beta (A*β*) and intracellular neurofibrillary tangles composed of protein tau as two of the major hallmarks and etiological factors [5].

Heat shock protein (HSP) is one kind of chaperones acting on the folding of proteins in normal metabolism and amplified the levels of repair and refolding of damaged polypeptides under stress. Hsp70 and Hsp90, the members of five main families of HSPs [6], are believed to play a profound role in tumor progression and response to therapy [7], which is also correlative with A*β* and protein tau. In this review, we will discuss the multiple functions of Hsp70 and Hsp90 in the occurrence and development of cancer and AD, focusing on proteins which regulated by HSP 70/90 and influence the occurrence and development of the two diseases.

## 2. HSP70/90: The Chaperones in the Formation and Stabilization of Protein Complexes

### 2.1. Hsp70 and Its Structure

The 70 kilodalton heat shock proteins (Hsp70s) are a kind of conserved ubiquitously expressed heat shock proteins that consist of eight homologous chaperones and play a fundamental role in the cell's machinery for protein folding [8, 9]. Six family members reside in the cytosol and nucleus, while the other two reside in the mitochondria and endoplasmic reticulum.

The structure of HSP70 consists of three domains: N-terminal ATPase domain that is the exchange of ATP which drives conformational changes in the other two domains; substrate binding domain that contains a groove with an affinity for neutral and ability to interact with peptides up to seven residues in length; C-terminal domain rich in alpha helical structure acts as a “lid” for the substrate binding domain which is open with ATP bound and closed with ADP bound.

### 2.2. The Functions of Hsp70

Hsp70, a protein unfolding machine, binds and releases stretches of hydrophobic amino acids in a regulated ATP-hydrolysis-driven cycle. Hsp70 is usually in an ATP bound state with very weak ATPase activity that spontaneous hydrolysis will not occur for many minutes, when not interacting with a substrate peptide. The substrate binding domain of Hsp70 recognizes and interacts with sequences of hydrophobic amino acid residues of newly synthesized proteins that emerge from the ribosomes. The peptides are freely bound and released by the ATPase of Hsp70. The ATPase activity of Hsp70 will be stimulated by the presence of a peptide in the binding domain, leading to slow rate of ATP hydrolysis. When ATP is hydrolyzed to ADP, the binding pocket of Hsp70 closes. Then Hsp70 is tightly binding the now-trapped peptide chain.

**Figure 1:**
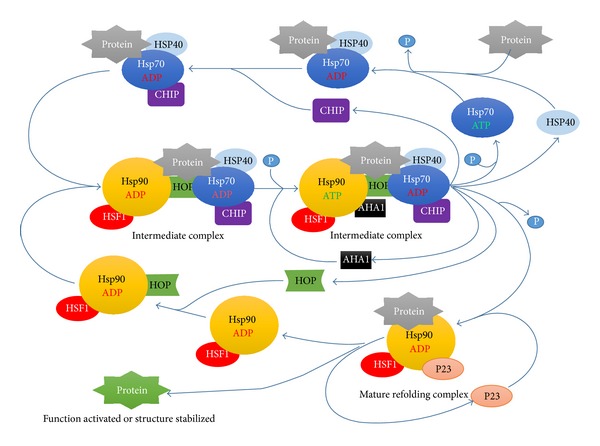
The Hsp70 and Hsp90 chaperone cycle. Protein is recognized by the Hsp70/40 complex with CHIP. The HOP facilitates transfer of the protein from Hsp70/40 complex to Hsp90 complex. Cochaperone p23 binds to complex of HSP90 and client protein to refold the client protein. Then the folded protein, HSP90, and cochaperone p23 release from the complex. Hsp70, Hsp40, HSP90, AHA1, CHIP, and p23 are regenerated that could be participated in the next cycle of protein folding action.

Hsp70 prevents the substrate from aggregating and being rendered nonfunctional by binding tightly to partially synthesized peptide sequences. When the protein is synthesized, a nucleotide exchange factor stimulates the release of ADP and binding of fresh ATP, opening the binding pocket. Then the protein is free to fold on its own, or to be transferred to other chaperones for further processing (Figure 1).

### 2.3. Hsp90 and Its Structure

HSP90, as a kind of chaperone proteins expressed by all eukaryotic cells, consists of four members including stress-induced HSP90*α*, constitutively expressed HSP90*β*, TNF receptor-associated protein 1 (TRAP1), and glucose-regulated protein 94(GRP-94) [10].

HSP90 consists of four structural domains: a highly conserved N-terminal domain (NTD) with ATPase activity; a middle domain (MD) with client protein binding functions; and a C-terminal domain responsible for the interaction with cofactors (dimerizing domain) [11–13].

### 2.4. The Functions of Hsp90

HSP90 functions form a multicomponent complex with cochaperones including Hsp40, Hsp70, Hop (Hsp70 and Hsp90 organizing protein), Cdc37, and p23 that serve to recognize client proteins and assist their binding to the Hsp90 heteroprotein complex [14, 15]. It is not entirely clear that the exact mechanism of the Hsp90 protein regulates the intracellular protein balance between folding and degradation. Based on the previous studies, the exposed hydrophobic amino acids of aberrant or misfolded client proteins bind to HSP40 and HSP70 to prevent aggregation. Then HSP70/40 passed the client protein to HSP90 assisted by Hsp70/Hsp90 organising protein (HOP). Cochaperone p23 bound to complex of HSP90 and client protein to refold the client protein. Then the folded protein, HSP90, and cochaperone p23 released from the complex. HSP90 and cochaperone p23 were regenerated that could be participated in the next cycle of protein folding action (Figure 1).

## 3. HSP70/90 in Cancer: Implicated in Many Crucial Steps in Tumorigenesis

HSP70/90 have been implicated in four of crucial steps of tumorigenesis including [16] (1) stabilizing oncogenic proteins [17, 18]; (2) inhibiting programmed cell death [19, 20] and replicative senescence [21, 22]; (3) induction of tumor angiogenesis [23–25]; (4) activation of invasion and metastasis [7, 26–28].

### 3.1. Stabilizing Oncogenic Proteins

Nearly 200 client proteins rely on the complex; HSP90 functions as part of it, for maturation and stability. Some of these client proteins participate in functions that promote cell growth, proliferation, and cell survival, which were associated with cancerogenesis [29]. Based on previous study, plenty of cancer associated proteins with primary structure change are unstable that can be restored to the native folding by HSP90 including [30–34] apoptotic mediators (Bcl-2, Apaf-1), tumor suppressor genes (Retinoblastoma protein, P53), cell cycle regulatory proteins (CDK4, hTERT), mediators of tissue invasion and metastasis (MMP2) [35], transcription factors (HSF-1, HIF-1), signaling molecules (SRC, LCK, AKT, RAF-1), steroid hormone receptors (androgen, progesterone, glucocorticoid receptors), and mutant fusion kinases (Bcr:abl, Nmp/akl) [36].

### 3.2. Inhibit Programmed Cell Death

There are multiple cytosolic pathways that Hsp90 inhibits cell from apoptosis. The formation of Hsp90-Apaf-1 complexes negatively regulates cytochrome c-mediated oligomerization of Apaf-1, which prevents the assembly of the apoptosome [20, 37]. A complex with Hsp90-Akt inhibits ASK1 (apoptosis signal-regulating kinase 1), a proapoptotic kinase [38]. Hsp90 inside mitochondria, with high level contained in cancer cells, also presents antiapoptosis function by interacting with TRAP1 (tumor necrosis factor receptor-associated protein 1), CYPD (cyclophilin D), and surviving [39]. It also prevents AIF (apoptosis-inducing factor) mitochondrial-cytosolic translocation and inhibits the nucleolytic activities of both AIF and endonuclease G [39]. Pharmacological targeting of HSP90 with specific chemical inhibitors leads to degradation of the client proteins and inhibition of tumor growth through G1 arrest, morphological and functional differentiation, and activation of apoptosis [23]. High levels of Hsp27 and Hsp70 are also considered as programmed cell death blocker by directly sequestering intermediates in the caspase-dependent apoptosis pathway [20].

### 3.3. Inhibit Replicative Senescence

A previous study reported that knockdown of Hsp72 in certain cancer cells triggers senescence via p53-dependent and p53-independent mechanisms [21]. The p53-dependent pathway controlled by Hsp72 depends on the oncogenic form of phosphatidylinositol 3-kinase (PI3K). Indeed, upon expression of the oncogenic PI3K, epithelial cells began responding to Hsp72 depletion by activating the p53 pathway. Moreover, in cancer cell lines, activation of the p53 pathway caused by depletion of Hsp72 was dependent on oncogenes that activate the PI3K pathway. On the other hand, the p53-independent senescence pathway controlled by Hsp72 was associated with the Ras oncogene. In this pathway, extracellular signal-regulated kinases (ERKs) were critical for senescence, and Hsp72 controlled the ERK-activating kinase cascade at the level of Raf-1. Importantly, upon Ras expression, untransformed cells started responding to knockdown of Hsp72 by constitutive activation of ERKs, culminating in senescence. Therefore, Hsp72 is intimately involved in suppression of at least two separate senescence signaling pathways that are regulated by distinct oncogenes in transformed cells, which explains why cancer cells become “addicted” to this heat shock protein [22].

### 3.4. Induction of Tumor Angiogenesis

In vascular endothelial cells, Hsp90 has been reported to associate with eNOS and participate in the regulation of eNOS activity [40]. NO generated by the eNOS has been shown to regulate endothelial cell growth and is essential for angiogenesis [41]. Hsp90 inhibitors reduce expression of VEGFR-1 on human vascular endothelial cells, VEGFR-3 on lymphatic endothelial cells in vitro, and all three VEGFRs on mouse vasculature in vivo [42].

### 3.5. Activation of Invasion and Metastasis

A previous study reported that HSP70/90 are overexpressed in a wide range of cancers and implicated in tumor invasion and metastasis [7], which can be considered as the most important poor prognosis factors. We summarized the relationship of expression level of Hsp70/Hsp90 and prognosis of the different kinds of cancer below.

Based on the past research, high level of Hsp70 expression is associated with poor prognosis in breast cancer [43–46], endometrial cancer [47, 48], uterine cervical cancer [48], and bladder cancer [49]. Contrarily Hsp70 expression was correlated with good prognosis in melanoma [50, 51], esophageal cancer [52–54], pancreatic cancer [55], and renal cancer [56]. Hsp70 expression showed no correlation with prognosis in ovarian cancer [56, 57], oral cancer [58], head and neck squamous cancer [59], gastric cancer [60], prostate cancer [61], and leukemia [62].

High expression of Hsp90 in cancer tissues is correlated with early recurrence and poor overall survival in breast cancer [63]. There is an association between the presence of autoantibodies to Hsp90 and poor prognosis in breast cancer [64]. In contrast, Hsp90 expression is associated with good prognosis in endometrial cancer [65]. In bladder carcinoma loss of Hsp90 expression is correlated with invasive recurrence risk [66].

As we can see, the correlations between Hsp70/90 and prognosis of cancer are very versatile and can change in different malignant tissues and in individual tumor cell populations. There can be confusion regarding the roles that Hsp70/90 play under individual circumstances. Actually there are great variations in the molecular mechanism of tumorigenesis and progression between the different kinds of cancer, even individuals. The proteins, regulated by Hsp70/90 may play a key role in promoting some kinds of cancer while without any correlation with the others.

As a consequence, Hsp70/90 implicates in many crucial steps in tumorigenesis and appears as biomarkers of cell differentiation (good and poor) or indicators of disease prognosis (good and poor).

## 4. Induction of HSP70/90 High Expression in Cancer

The mechanisms of the induction of HSP70/90 in cancer are not fully clear. Heat shock factor 1 (HSF1), which consists of Hsp90-containing HSF1 complex in the unstressed cell and dissociates during stress, was the key to activate HSP gene expression [67]. Stress induces HSF1 into homotrimer, and a number of posttranslational modifications (PTM) that convert HSF1 into an active form. Then HSF1 moves toward a nuclear localization and binds the promoter of HSP genes to stimulate the HSP gene expression [68, 69]. Hence, HSF1 play a crucial role in regulation of the level of HSPs, which is regulated by some of tumor-related genes and proteins. HSP elevation in tumor cells can be induced by the highly malignant factor HRG*β*1, which is a secreted factor that binds to c-erbB receptors and induces HSP expression through HSF1 [19]. Inactivation of the HSF1 gene prevents HSP induction by HRG*β*1. HSP expression is induced through a cascade response initiated by HRG*β*1 on the cell surface and which leads to the inhibition of intracellular HSF1. SUMoylation, a PTM observed frequently in transcription factors that are associated with carcinoma, is also associated with HSF1 [70]. The deacetylase sirtuin-1, a factor associated with cancer, promotes deacetylation of HSF1 to activate it [71]. In cancer, gene promoter CpG islands acquire abnormal hypermethylation, which results in transcriptional silencing that can be inherited by daughter cells following cell division [72]. The HSF1 gene also contains a number of CpG dinucleotides that could lead to its silencing under some conditions [73]. Further research of the roles of HSF1 in Induction of HSP70/90 high expression in cancer is needed.

## 5. HSP70/90 in Alzheimer's Disease: Protein Stabilizer and Regulator

Extraneuronal deposits of A*β* and intracellular neurofibrillary tangles composed of the protein tau, which are pathological hallmark and etiological factor of AD, are reported to have correlation with disfunction of inducing form of HSP70/90.

### 5.1. Hsp70: Inhibitive Factor of AD

The Hsp70, as a protein stabilizer, has a cellular protection against neurodegeneration of the central nervous system which is proved by several animal models [74–77]. Previous studies have showed that the functional disorder of Hsp70 related to neuroprotective system play an important role in development of AD [78], and intranasally administered Hsp70 enters the afflicted brain regions and mitigates multiple AD-like morphological and cognitive abnormalities observed in model animals [5]. Extraneuronal deposits of A*β* and intracellular neurofibrillary tangles composed of the protein tau, which are pathological hallmark and etiological factor of AD, are reported to have correlation with disfunction of inducing form of HSP70. Then, we intend to thoroughly discuss the relationship between Hsp70 and AD according to the result of researches.

Hsp70 protect against the toxic effects of A*β* accumulation by interfering in A*β* homeostasis. AD is distinguished histopathologically from other dementias by abundant extraneuronal deposits of A*β*. The major pathological hallmark of AD plays an early and important pathologic role in the development of AD. Several studies proposed that soluble A*β* oligomers extracted from AD brains potently impair synapse structure and function. These A*β* peptides are toxic in oligomeric forms and have a tendency to form fibrils and to aggregate in the brain, eventually depositing as plaques, which is a crucial event in AD [79]. Many previous studies widely accepted that A*β* aggregates trigger a series of downstream events such as plaque deposition, tau hyperphosphorylation, inflammation, loss of synaptic structure and function, and death of susceptible neurons, which was deemed as “amyloid cascade hypothesis” [80–83]. The wealth of evidence from many independent investigators worldwide supported that A*β* dyshomeostasis played a central role in AD pathogenesis [84]. So, Hsp70 plays an important role in AD pathogenesis by inhibition of A*β* oligomerization and enhancing the clearance of A*β*. Several studies have demonstrated that HSP70 overexpression effectively protected neurons from accumulation of *А* *β* in AD [85, 86]. HSP70 recognized A*β* oligomers and decrease the level of A*β* self-assembly, resulting in the suppression of the production of toxic A*β* [77, 87, 88]. HSP70 also stimulated the degradation of A*β* by enzyme mediated degradation, phagocytosis by microglia, and astrocytes [89]. HSP70 promote the stimulation of A*β* clearance through upregulation of expression of insulin degrading enzyme (IDE) and TGF-*β*1. IDE is an A*β*-degrading enzyme that motivates the clearance of A*β*. TGF-*β*1 was a key cytokine regulating the response of the brain to injury and inflammation has also been suggested to suppress the progression of AD. TGF-*β*1 stimulates A*β* clearance through activation of phagocytic microglia.

**Figure 2:**
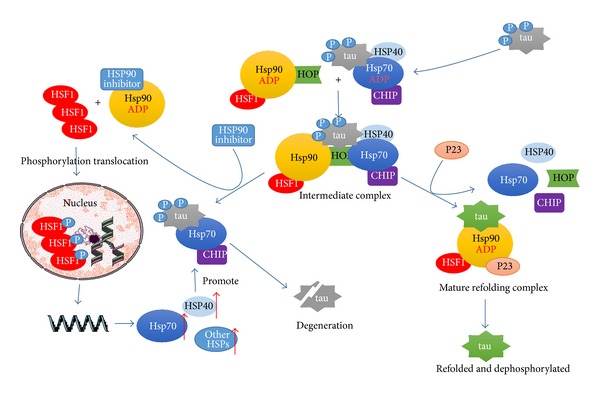
The role of Hsp70 and Hsp90 chaperone in the regulation of tau protein. The dephosphorylation and refolding of protein tau is facilitated by Hsp70/90 complex, preventing degradation; when the Hsp90 inhibition is initiated, tau is transferred to the Hsp70/CHIP complex undergoing degradation. The inhibition of Hsp90 promotes the depolymerization of Hsp90-containing HSF1 complex and induces HSF1 into homotrimer to activate HSP gene expression.

Hsp70 plays cytoprotective roles in AD by maintaining tau homeostasis. Neurofibrillary tangles (NFTs) composed of aggregates of hyperphosphorylated forms of the protein tau are considered as another major histopathological character in AD. Tau protein, one of type II microtubule-associated proteins (MAPs), plays a key role in maintenance and stabilization of microtubules within axons in AD. The aggregation and accumulation of protein tau are believed to be responsible for AD pathogenesis [90]. Hsp70 is able to form complexes with tau, implicate in blocking tau aggregation, and be involved in its degradation process [91, 92]. Hsp70 inhibits tau aggregation by a mechanism involving preferential associations with soluble, monomeric, and prefibrillar oligomeric tau species and assists these combinations being degraded by the ubiquitin-proteasome and autophagy system (Figure 2) [93]. In a cell-based model, inhibiting Hsp70 ATPase activity after increasing its expression levels also facilitated tau degradation [94].

### 5.2. HSP90

Hsp90 maintains the functional stability of neuronal proteins of aberrant capacity, thus allowing and sustaining the accumulation of toxic aggregates [95, 96]. Hsp90 has two aspects of roles: a crucial chaperone consists of the complex that regulate the tau protein; a repressor of heat shock transcription factor-1 (HSF1), which binds to upstream regulatory sequences in the promoters of heat shock genes [67, 97].

### 5.3. Hsp90 Complexes: Chaperone-Assisted Tau Degradation

The main function of Hsp90 complexes is to maintain protein quality control and assist in protein degradation via proteasomal and autophagic-lysosomal pathways [98]. The Hsp90 complexes have the ability to recognize misfolded proteins and assist in their conversion to a functional conformation or guide them towards proteasomal degradation [99–101].

Hsp90 complex, in collaboration with CHIP protein, plays a key role in the removal of phosphorylated tau protein [102]. First, ubiquitin tag needs to be labeled to tau proteins, by carboxy terminus of Hsc70 interacting protein (CHIP) with U-box domain contains its E3 ubiquitin ligase activity, which also interacts with Hsp90 complex through its TPR domain [103]. Then E3 ubiquitin ligase of CHIP interacts with specific ubiquitin conjugating E2 enzymes, which linked to K48 or K63 ubiquitination to control whether the misfolded protein will be directed to proteasomes or aggresomes [104–106]. Tau protein is a client protein for these Hsp90 complexes. If the tau protein is in an abnormal or modified form, then it can trigger the recruitment of CHIP protein, a cochaperone with E3 activity, to the complex which induces the ubiquitination of tau protein and activates its downstream degradation processes (Figure 2).

### 5.4. Hsp90 Regulate the Expression of HSPs

Hsp90, by itself and/or associated with multichaperone complexes, is a major repressor of heat shock transcription factor-1 (HSF1), which binds to upstream regulatory sequences in the promoters of heat shock genes [67, 97]. Namely, under nonstressed conditions, Hsp90 binds to HSF-1 and maintains the transcription factor in a monomeric state [95]. Stress or inhibition of Hsp90 induces dissociation of Hsp90/HSF-1 complex, while HSF-1 translocates to the nucleus and initiates the production of Hsps such as the chaperones Hsp70 and its activator, Hsp40 (Figure 2).

In AD brains, levels of Hsp90 were increased in both cytosolic and membranous fractions, and Hsp90 was colocalized with amyloid plaques [107]. We speculate that Hsp90 promote neurodegenerative disorders indirectly through regulating the expression of Hsp70 and other chaperones.

## 6. Conclusion

Hsp70/90 are inducted by stress to amplify the levels of repair and refolding of damaged polypeptides and participate in maintaining the stability of proteins, which play substantial roles in both carcinogenesis and pathogenesis of AD [108]. HSP70/90 have been implicated in four of crucial steps of carcinogenesis: stabilizing oncogenic proteins, inhibiting programmed cell death and replicative senescence, induction of tumor angiogenesis, and activation of invasion and metastasis. The Hsp70, as a protein stabilizer, has a cellular protection against neurodegeneration of the central nervous system. However, Hsp90 promotes neurodegenerative disorders indirectly through regulating the expression of Hsp70 and other chaperones. These make the Hsp70/90 exciting targets in cancer and AD. Unfortunately, the correlations between Hsp70/90 and prognosis of cancer are very versatile and can change in different malignant tissues and individuals. High expression of them indicates different prognoses even in the same kind of disease. This phenomenon can be attributed to the complexity of oncogenesis. A series of several mutations to certain classes of genes is usually required before a normal cell will transform into a cancer cell. Hence, multiple mutated proteins act on different intracellular signal transduction pathways to promote or restraint the proliferation of cancer cells. Regulation effect of Hsp70/90 on the different proteins can lead to crucially different outcomes. Meantime, AD is a complex neurodegenerative disease involving the interactions among various potential biological and environmental factors. It is difficult to measure whether Hsp70/90 play the most crucial role in the development of AD. Existing evidences showed Hsp70/90 as therapeutic targets in cellular and animal models have some limitations must be overcome before clinical application in human. The clinical uses of Hsp70/90 as drug targets need further efforts to investigate the mechanisms of them.
